# A transient window of hypothyroidism alters neural progenitor cells and results in abnormal brain development

**DOI:** 10.1038/s41598-019-40249-7

**Published:** 2019-03-15

**Authors:** Katherine L. O’Shaughnessy, Susan E. Thomas, Stephanie R. Spring, Jermaine L. Ford, Richard L. Ford, Mary E. Gilbert

**Affiliations:** 1 U.S. Environmental Protection Agency, National Health and Environmental Effects Research Laboratory, Toxicity Assessment Division, Endocrine Toxicology Branch, Research Triangle Park, North Carolina, NC 27711 USA; 2 Oak Ridge Institute for Science Education, Oak Ridge, TN 37830 USA; 3 U.S. Environmental Protection Agency, National Health and Environmental Effects Research Laboratory, Analytical Chemistry Core, Research Triangle Park, North Carolina, NC 27711 USA

## Abstract

Cortical heterotopias are clusters of ectopic neurons in the brain and are associated with neurodevelopmental disorders like epilepsy and learning disabilities. We have previously characterized the robust penetrance of a heterotopia in a rat model, induced by thyroid hormone (TH) disruption during gestation. However, the specific mechanism by which maternal TH insufficiency results in this birth defect remains unknown. Here we first determined the developmental window susceptible to endocrine disruption and describe a cellular mechanism responsible for heterotopia formation. We show that five days of maternal goitrogen treatment (10 ppm propylthiouracil) during the perinatal period (GD19-PN2) induces a periventricular heterotopia in 100% of the offspring. Beginning in the early postnatal brain, neurons begin to aggregate near the ventricles of treated animals. In parallel, transcriptional and architectural changes of this region were observed including decreased Sonic hedgehog (*Shh)* expression, abnormal cell adhesion, and altered radial glia morphology. As the ventricular epithelium is juxtaposed to two sources of brain THs, the cerebrospinal fluid and vasculature, this progenitor niche may be especially susceptible to TH disruption. This work highlights the spatiotemporal vulnerabilities of the developing brain and demonstrates that a transient period of TH perturbation is sufficient to induce a congenital abnormality.

## Introduction

Normal brain development requires thyroid hormone (TH) during both pregnancy and the postnatal period in humans^1^. This dependence is classically represented by cretinism, where severe growth and intellectual disability are observed in children experiencing TH dysfunction during development, like in cases of maternal iodine deficiency^2^ or congenital hypothyroidism^3^. More recently several epidemiological studies have also identified associations between maternal thyroid hormone insufficiency and neurodevelopmental outcomes in children; these include cognitive deficits^4–7^, attention deficit hyperactivity disorder (ADHD)^8^, and increased seizure susceptibility^9^. Given the established link between TH homeostasis and the brain, identifying the precise mechanisms by which TH disruption impacts neurodevelopment has direct public health implications. This extends not only to the clinical management of thyroid conditions in pregnant mothers, but also to interpreting the risks of environmental endocrine disrupting chemicals. Many environmental toxicants have been identified that interfere with the synthesis, transport, and/or function of thyroid hormones, all which could result in TH dysregulation^10^. These manufactured chemicals, referred to as thyroid toxicants, include components of some pesticides, plastics, and oxidizers (notable thyroid toxicants reviewed in Gore *et al*. 2015). However, many challenges remain in understanding the effects of thyroid toxicants on the mother and child.

**Figure 1:**
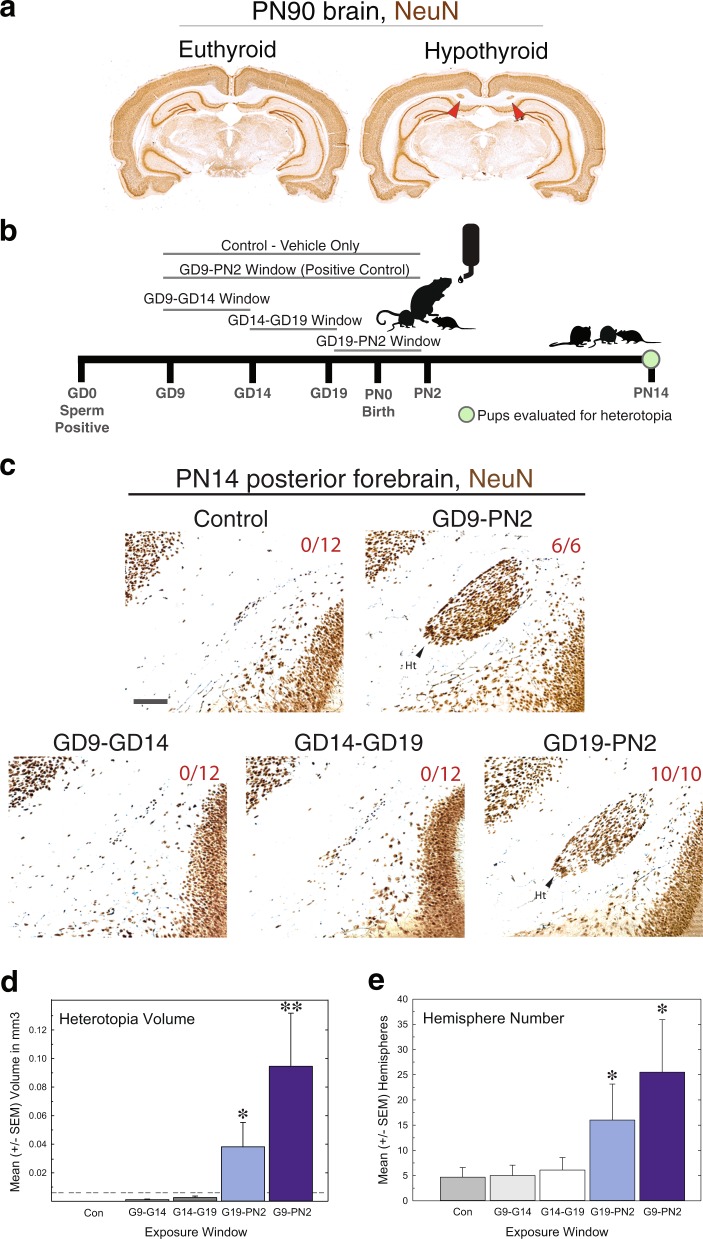
Perinatal exposure to an anti-thyroid agent is sufficient for cortical heterotopia formation. (**a**) The heterotopia is a permanent malformation. Light micrographs depict coronal brain sections from adult male rats born to either control (euthyroid, left) or PTU treated dams (hypothyroid, right). The heterotopia is visualized by Neuronal Nuclei (NeuN) immunostaining (red arrow). (**b**) The experimental design for determining the developmental period necessary for heterotopia formation. Timed pregnant dams were treated with 10 ppm PTU via the drinking water over one of three windows (GD9-GD14, GD14-GD19, and GD19-PN2). A positive control group was also included (dams treated GD9-PN2). Control animals received plain drinking water only. Offspring were analyzed on PN14 for heterotopia presence and severity. (**c**) Representative sections on posterior forebrain on PN14. Only animals from the positive control (GD9-PN2) and perinatal (GD19-PN2) treatments possessed this birth defect. The numbers on each panel represent the number of animals screened with a heterotopia; one male and one female pup was analyzed per litter. Scale bar represents 200 µm. (**d**) As there is no sex difference in heterotopia formation^14^, the calculated heterotopia volume from the male and female littermates were averaged before analysis. The dashed line represents the minimum criterion for a neuron cluster to be considered a heterotopia (volume ≥ 0.006^3^ mm). Pups born to dams treated from GD9-PN2 and GD19-PN2 are significantly different than controls. (**e**) Analysis of the number of hemispheres (both right and left) in which ectopic neurons were observed in the heterotopia forming region across a 1.8 mm anterior-posterior interval of the brain. In panels (d) and (e) error bars represent ± SEM and asterisks p < 0.05. N = 3–6 litters analyzed.

One of the major difficulties in understanding how TH toxicants impact brain development and function is the lack of robust phenotypes resultant from mild/moderate hormone disruption in animal models. While severe insults to the thyroid axis using thyroidectomy and/or high doses of anti-thyroid agents have been explored (reviewed in Bernal, 2015)^11^, these results may not be recapitulated in the context of a milder hormone perturbation that is more representative of human conditions. Of the neurodevelopmental phenotypes described in hypothyroid rodent models, one that is highly reproducible and dose-dependent is a subcortical band heterotopia^12–15^ (Fig. 1a). Characterized as clusters of ectopic neurons within the corpus callosum, this permanent birth defect is observable in rat offspring born to both hypothyroid and TH insufficient dams. Interestingly, this phenotype is present in offspring even when maternal thyroid stimulating hormone (TSH) is not significantly increased^13^, an intriguing observation given that TSH is the benchmark measure for estimating TH dysfunction during human pregnancy. While we currently understand that the heterotopia is ameliorated by maternal T4 infusion^12^, and that primarily prenatal TH insufficiency is required for its formation^14^, we do not know what cellular processes underlie its development.

To address when and how maternal TH disruption leads to this structural defect of the brain, we first identified the developmental window susceptible to hormone insufficiency. Timed pregnant rats were administered a low dose of the anti-thyroid pharmaceutical propylthiouracil (PTU) across four discrete gestational windows, and offspring were analyzed for heterotopia on postnatal day (PN) 14. We show that five days of maternal exposure from gestational day (GD) 19 – PN2 is sufficient for complete penetrance of the cortical heterotopia in pups; no other exposure window of similar duration induced this phenotype. Examination of serum and brain THs demonstrates that this perinatal exposure reduces THs during the first postnatal week in pups. In parallel with published genetic models of heterotopia, we show abnormal polarity of radial glial cells and alterations in the integrity of the ventricular epithelium in affected neonates. We postulate that insufficient adhesion at the apical surface of the ventricular epithelium compromises the normal attachment of radial glial end feet, leading to disorganization of the radial glial fibers. This disruption of the migratory scaffolding promotes aggregation of neurons near the lateral ventricles, resulting in heterotopia. Furthermore, we propose that the ventricular epithelium of the developing brain is acutely sensitive to TH dynamics. This is due to (i) its expression of the TH mediator sonic hedgehog (SHH), and (ii) its proximity to the cerebrospinal fluid and vasculature, the two sources of brain THs. This work highlights the spatiotemporal susceptibilities of the developing central nervous system and suggests that the timing of TH insufficiency is a critical variable when interpreting the risk of TH disruption during pregnancy.

## Results

### Exposure to an anti-thyroid agent during the perinatal period is sufficient for heterotopia formation in rats

To identify the developmental period susceptible to TH disruption, pregnant rats were treated with a low dose of PTU (10 ppm) via the drinking water over four distinct gestational windows (summarized in Fig. 1b). The first maternal dosing window spanned from GD9 and persisted until PN2; this exposure served as a positive control, in which all offspring are expected to possess cortical heterotopia (N = 3 dams)^14^. The three remaining treatment paradigms were shorter in duration, and spanned GD9-GD14, GD14-GD19, and GD19-PN2 (N = 5–6). Control animals received deionized drinking water (N = 6). On PN14, one male and one female pup from each litter were assayed for heterotopia presence (see methods for criterion). Results demonstrate that maternal PTU treatment from GD19-PN2, the perinatal period, is both sufficient and necessary to induce a cortical heterotopia in 100% of the offspring analyzed on PN14 (Fig. 1c, 10/10 animals, two littermates were assayed across five litters). No animals from any other exposure possessed this birth defect, albeit pups born to dams treated with PTU from GD9-PN2, which represents the positive control treatment (Fig. 1c, 6/6 animals, two littermates were assayed across three litters). Analysis of average heterotopia volume demonstrates that pups from the GD9-PN2 and the GD19-PN2 exposures were significantly different from controls [F(4,21) = 29.63, p < 0.0001] (Fig. 1d). It is noted that the severity of this defect is exacerbated in animals from the GD9-PN2 exposure, as demonstrated by an average 125% increase in heterotopia volume as compared to the five-day treatment (Fig. 1d, compare the GD19-PN2 and GD9-PN2 data). Quantification of the number of hemispheres in which a cluster of ectopic neurons was observed in the heterotopia forming region (Fig. 1a, within the corpus callosum, inferior to the cingulum), further demonstrates that animals from the GD9-PN2 and GD19-PN2 treatments exhibit a greater incidence of aggregated neurons within the white matter tract (Fig. 1e).

### Perinatal exposure did not induce overt toxicity

**Figure 2:**
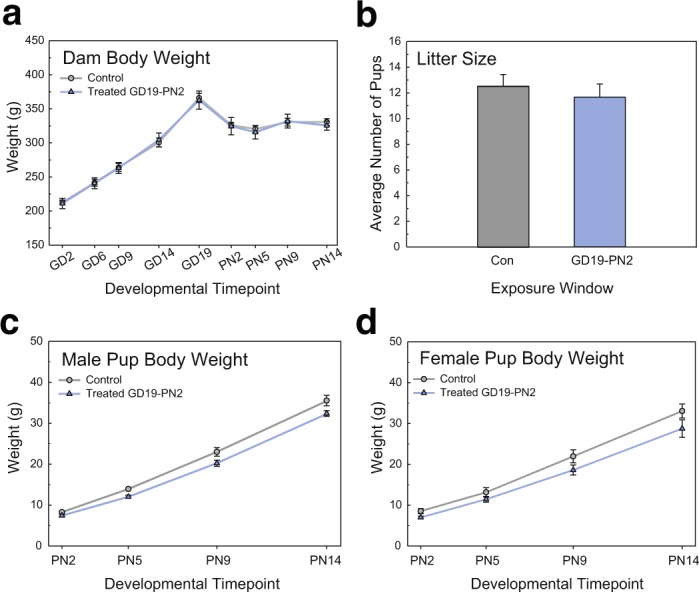
Maternal PTU treatment during the perinatal period did not induce overt developmental toxicity. (**a**) Body weight in dams treated from GD19-PN2 with 10 ppm PTU was not significantly altered as compared to controls. (**b**) Litter size (recorded on PN2) was similarly unaffected. (**c**) Male and (**d**) female pup body weight was slightly, but not significantly, reduced during the first two postnatal weeks. In all analyses error bars represent ± SEM and asterisks p < 0.05. N = 5–6 litters per treatment analyzed.

To determine whether the PTU treatment induced overt developmental toxicity, dam body weight, litter size, and pup body weights were evaluated. The five day perinatal exposure, while inducing a highly penetrant cortical malformation in the developing animal, produced no significant alterations in dam body weight during the pre- or postnatal period [N = 6 controls, N = 5 treated, F(3,19) = 0.50, p = 0.6879] (Fig. 2a). Litter size of dams treated from GD19-PN2 was also not significantly different as compared to controls [N = 6 controls, N = 5 treated, t(0) = 0.77, p = 0.4636] (Fig. 2b). Neither male nor female pup body weight was significantly affected by maternal PTU treatment [N = 6 controls, N = 5 treated, F(1,8) = 4.12, p = 0.0769 and F(1,9) = 2.77, p = 0.1306, respectively] (Fig. 2c,d). Together, these data show that maternal PTU exposure from GD19-PN2 did not induce gross developmental toxicity, despite 100% penetrance of the cortical heterotopia.

### Serum and brain THs are transiently reduced in response to maternal PTU treatment

**Figure 3:**
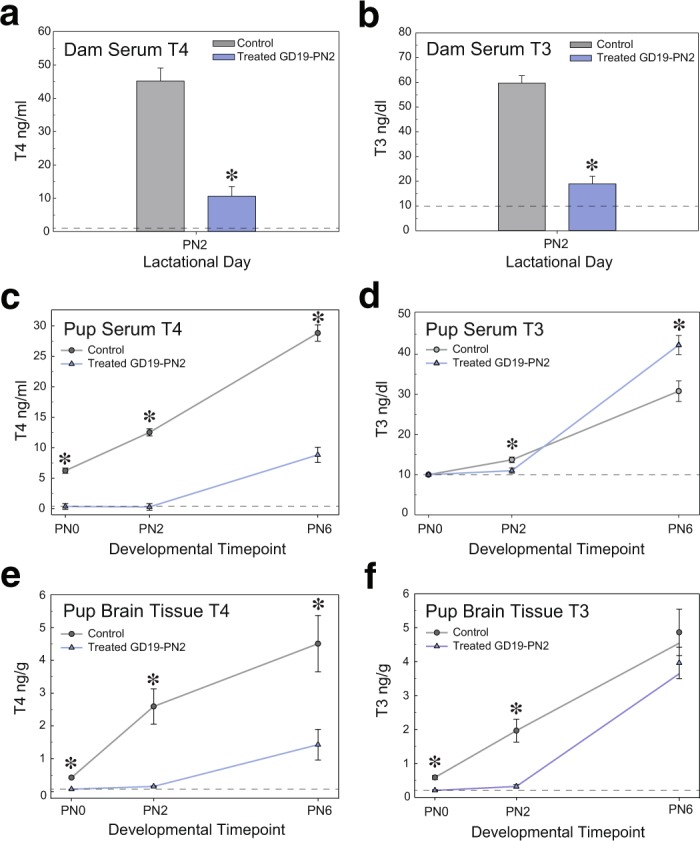
Five days of maternal PTU treatment reduces serum T4 and T3 in both dams and pups. (**a**) Dams treated with PTU from GD19-PN2 exhibited a significant decrease in serum T4 and (**b**) T3 on PN2. (**c**) Pup serum T4 was significantly reduced in pups on PN0, PN2, and PN6. (**d**) Pup serum T3 was also significantly reduced on PN2; however, serum T3 was significantly elevated in treated pups as compared to controls on PN6. PN0 pup serum was sampled, but T3 concentrations were below the lower limit of quantification (LLOQ). (**e**) Pup brain T4 was significantly reduced in treated animals on PN0, 2 and 6. (**f**) Pup brain T3 concentrations, in comparison, were reduced in treated pups on PN0 and 2. However on PN6, T3 levels were not significantly different from controls. In all panels dashed lines denote the LLOQ for serum (0.1 ng/ml for T4 and 10 ng/dl for T3) and brain tissue (0.1 ng/g for both T4/T3). In all analyses error bars represent ± SEM and asterisks p < 0.05. N = 5–7 analyzed.

On PN2 total T4 and T3 concentrations were significantly reduced by approximately 70% in dams treated with PTU from GD19-PN2 [N = 6 controls, N = 5 treated, t(8) = 6.83, p < 0.0001 and t(8) = 7.21, p < 0.0001, respectively] (Fig. 3a,b). Serum total T4 concentrations in treated pups were significantly reduced by at least 74% in pups on all days assayed (PN0, 2, and 6), despite termination of PTU via the dam’s drinking water on PN2 [N = 6 controls, N = 5 treated, F(5,30) = 135.29), p < 0.0001] (Fig. 3c). In contrast, serum total T3 concentrations were significantly reduced in pups on PN0 and PN2, and significantly elevated (33%) when compared to controls animals on PN6 [N = 6 controls, N = 7 treated, F(5,30) = 78.90, p < 0.0001] (Fig. 3d). Pup serum TSH was also significantly elevated on PN2 and PN6 [N = 6 controls, N = 7 treated, F(3,21) = 48.89, p < 0.0001] (Supplementary Fig. 1). Similarly to what is observed in sera, pup brain T4 concentrations were significantly reduced at all ages evaluated [F(5,29) = 14.43, p < 0.0001] (Fig. 3e), whereas brain T3 was reduced only on PN0 and PN2 [F(5,29) = 25.57, p < 0.0001] (Fig. 3f).

### The heterotopia is periventricular and is comprised of late born neurons

**Figure 4:**
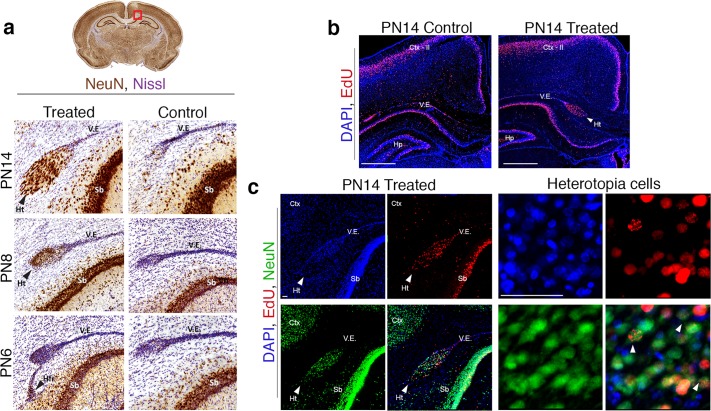
The heterotopia is periventricular. (**a**) Representative posterior forebrain sections in pups born to dams treated from GD19-PN2 (treated) and control animals. Coronal sections are stained with NeuN to detect mature neurons, and counterstained with Nissl. The cortical heterotopia reproducibly forms directly medial to the ventricular epithelium (V.E.), and superior to the subiculum (Sb). On PN6 aggregates of cells are observed in the treated animal in the heterotopia forming region (Hfr, black arrow); by PN8 NeuN+ cells begin to condense in the treated animal, representing the early neuronal heterotopia (Ht, black arrow). On PN14, the periventricular heterotopia is readily observable. (**b**) When PTU exposed dams are pulsed with the thymidine analog EdU on GD18–19, EdU labeling is detected in the pup heterotopia (Ht) on PN14 (white arrow, treated animal). Other cells born on GD18 and 19 contribute to the layer II of the neocortex (Ctx-II), the hippocampus (Hp), and the ventricular epithelium (V.E) of both treated and control animals. Scale bar represents 500 µm. (**c**) A portion of these late-born cells contributing to the heterotopia are neurons, as observed by co-labeling of EdU and NeuN. Scale bars represent 50 µm.

To examine the ontogeny of heterotopia development, the posterior forebrain was examined in control and treated pups during early postnatal development. This encompasses the period during, and immediately following, the observed significant reduction in brain T3. Neuronal nuclei (NeuN) immunohistochemistry in conjunction with Nissl counterstaining revealed the accumulation of cells near the lateral ventricle in PTU-treated pups on PN6 (Fig. 4a). By PN8 mature neurons (NeuN+) begin to condense directly medial to the lateral ventricle, and by PN14 a fully formed cortical heterotopia presents in treated animals (Fig. 3a). A stage series of Nissl stained sections in control tissue further illustrates that the heterotopia forms near the lateral ventricles of the posterior forebrain (Supplementary Fig. 2). At birth the lateral ventricles are clearly open, and the surrounding neuroepithelium distinct. However, clear morphological changes occur during the first postnatal weeks and by PN14, the ventricular epithelium has retracted into a thin layer within the dorsal corpus callosum (Supplementary Fig. 2). As our initial investigations of the heterotopia were primarily performed in animals from PN14-adulthood, this led to the non-specific characterization of the heterotopia as subcortical band as opposed to periventricular^12–15^. Thus, the anatomical positioning of the heterotopia is fully revealed by examination of ventricular structure during the early postnatal period (Fig. 4a and Supplementary Fig. 2).

Previous work has shown that the TH-induced heterotopia is comprised of neurons born during late gestation; however, this work was done under a prolonged PTU treatment spanning from early gestation to weaning (PN21)^12^. To confirm that the periventricular heterotopia is comprised of late born neurons, a subset of control and PTU treated dams were pulsed with the thymidine analog 5-ethynyl-2′-deoxyurdine (EdU) on GD18 and GD19 (Supplementary Fig. 3). Subsequent analysis of the PN14 pup brain demonstrates that cells contributing the heterotopia are indeed born on GD18-19, as evidenced by EdU labeling (Fig. 4b). Pronounced EdU labelling was also observed in the neocortex (predominantly layer II), the subiculum, and the hippocampus (CA1, CA3, and dentate gyrus) of both treated and control animals (Fig. 4b). Colocalization of EdU and NeuN labeling within the PN14 heterotopia verifies that a subset of these late born cells are mature neurons (Fig. 4c), and confirms the original observations reported in Goodman and Gilbert, 2007.

### Sonic hedgehog expression is reduced in hypothyroid animals

**Figure 5:**
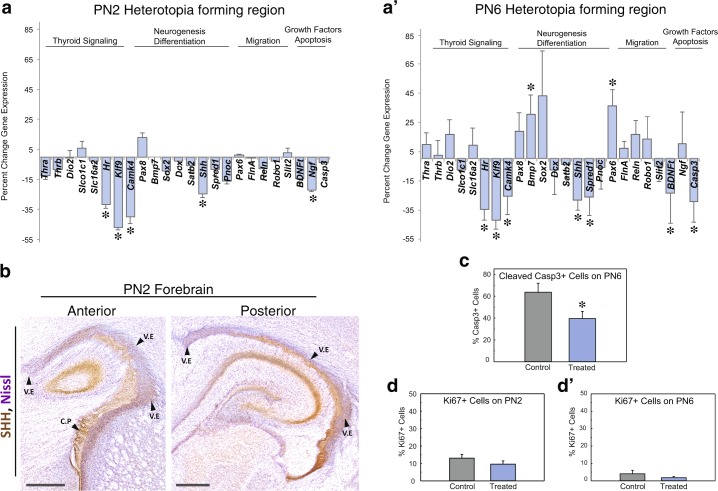
Sonic hedgehog (*Shh*), a direct TH target, is reduced in the postnatal brain following perinatal PTU exposure. (**a**) On PN2, a stage where T4/T3 are reduced in brain tissue, several genes associated with thyroid signaling are significantly downregulated. This includes *Shh*, a direct T3 target implicated in brain morphogenesis and stem cell maintenance. (**a’**) On PN6, *Shh* is also downregulated. However, eight more genes were differentially expressed in the posterior forebrain including sprouty related EVH domain 1 (*Spred1)*. Downregulation of *Spred1* in the perinatal mouse brain is associated with periventricular heterotopia formation. Asterisks in the gene expression data represent p < 0.001. N = 6–7 were analyzed at both stages. (**b**) SHH protein is normally expressed within the early postnatal ventricular epithelium (V.E.), hippocampus, and choroid plexus (C.P.) in control animals on PN2; scale bars represent 500 µm. **(c)** As we detected a downregulation in *Casp3* gene expression in the treated brain (see **a’**), we assayed for potential differences in cleaved-CASP3 by immunohistochemistry on PN6. PTU treated pups exhibited a significant decrease (p < 0.05) in the percentage of labeled cleaved-CASP3 cells in the heterotopia forming region. N = 6 were assayed. (**d**) No differences in the percent labeling of Ki67 positive cells were detected in the ventricular epithelium on PN2 or (**d’**) on PN6. In all panels error bars represent ± SEM and N = 4–7 were analyzed.

To investigate a potential cellular mechanism linking TH dysregulation to heterotopia development, we next performed targeted gene expression analyses in the posterior forebrain (see dissection summary, Supplementary Fig. 4). Specifically, molecules associated with TH signaling, neurogenesis, cell migration, and apoptosis were assayed. On PN2, a day of development in which both T4 and T3 are significantly reduced in brain tissue (Fig. 3e and f), we identified several genes that were differentially expressed in PTU treated animals (Fig. 5a, N = 6 controls and N = 7 treated assayed). These includes hairless (*Hr*), Kruppel-like factor 9 (*Klf9*), calcium modulated kinase VI (*Camk4*), sonic hedgehog (*Shh*), and nerve growth factor (*Ngf*). On PN6, the stage where neurons can be first observed accumulating near the lateral ventricular epithelium (Fig. 4a), *Hr*, *Klf9*, and *Shh* mRNA remain significantly downregulated (Fig. 5a’). In addition, several other genes were differentially expressed. These include downregulation of caspase 3 (*Casp3*), an intermediate of cell apoptosis^16^, downregulation of sprouty protein with EVH domain 1 (*Spred1)*, which is associated with periventricular heterotopia formation in mice^17^, and upregulation of paired box 6 (*Pax6)*, a marker of radial glial progenitor cells^18^ (Fig. 5a’). Given that (i) *Shh* is a direct mediator of T3 action in the brain^19–22^, and (ii) was downregulated in both PN2 and PN6 animals, we next investigated the normal distribution of this protein by immunohistochemistry. Localization of SHH in the PN2 posterior control forebrain reveals enriched expression throughout the ventricular epithelium in both the anterior and posterior forebrain, as well as within the cells of the choroid plexus (Fig. 5b).

### Cell death, but not proliferation, is significantly altered in the hypothyroid brain

As the heterotopia is characterized as ectopic neurons, we next tested whether changes in either cell death and/or cell proliferation within the ventricular epithelium may contribute to its formation. Immunohistochemistry of cleaved-CASP3 revealed a significant decrease in the percent of positively labeled cells in PN6 animals exposed perinatally to PTU [N = 6 control and N = 6 treated, t(10) = 2.29, p = 0.0454] (Fig. 5c). This result is complementary to the gene expression analyses, which also identified a significant downregulation in *Casp3* on PN6 (Fig. 5a’). Together these two analyses indicate a significant reduction in apoptosis within the heterotopia forming region of hypothyroid animals. Investigation of cell proliferation between control and treated animals identified no significant changes in the percent labeling of Ki67+ cells on PN2 [N = 7 control and N = 4 treated, t(9) = 1.00, p = 0.341] or PN6 [N = 4 control and N = 4 control, t(6) = 1.06, p = 0.332] (Fig. 5d and d’).

### Radial glial progenitors are disorganized in conjunction with TH dysregulation

**Figure 6:**
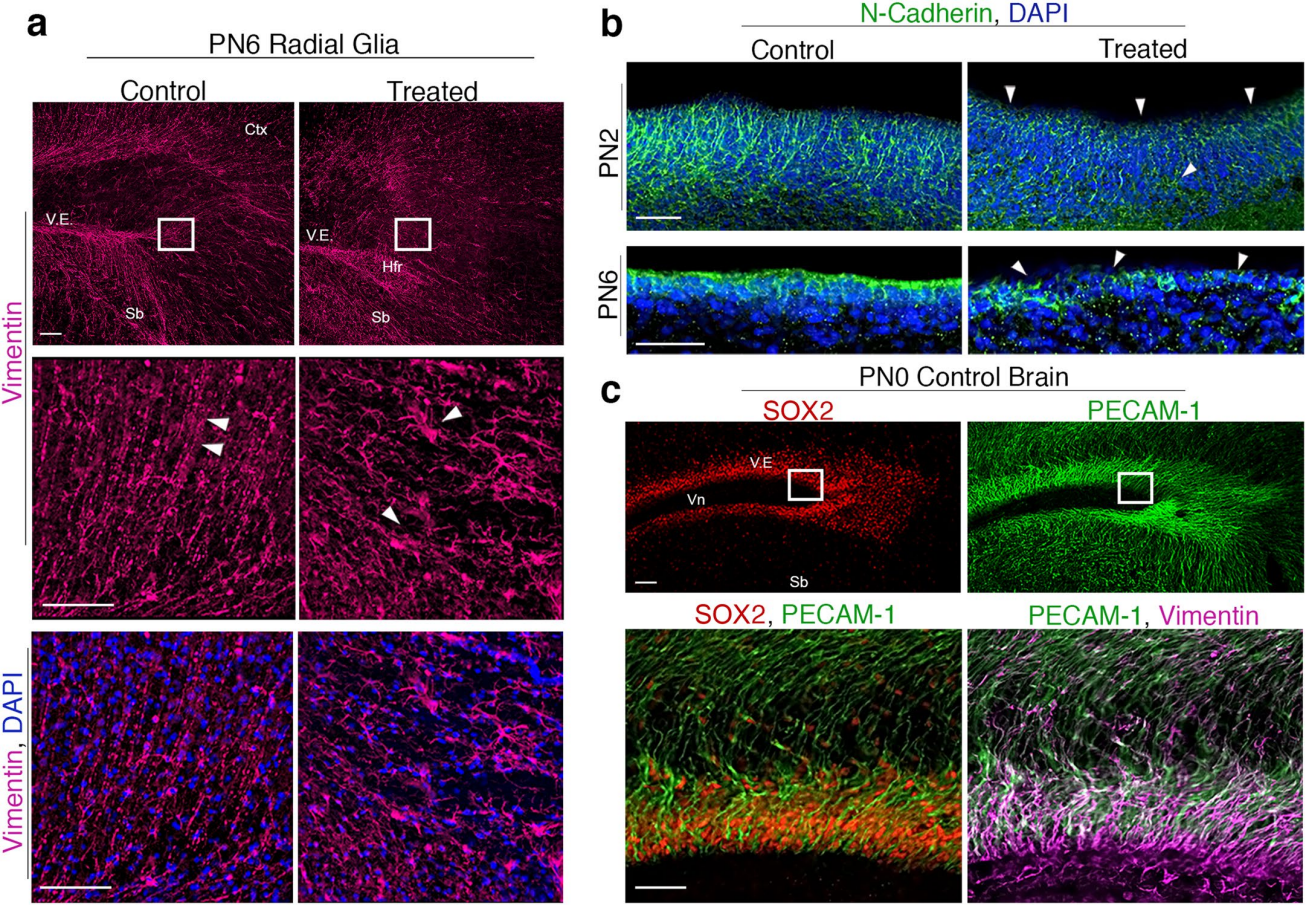
Radial glial progenitors and the ventricular neuroepithelium are altered in TH insufficient pups. (**a**) Radial glial progenitor cells in control and PTU treated animals on PN6. In control animals, the normal apico-basal polarity of these cells is observed. However, in the animals treated with PTU from GD19-PN2, radial glial cells appear truncated and highly disorganized in the heterotopia forming region (Hfr), the cortex (Ctx), and near the subiculum (Sb). The extent of these abnormalities is further observed at higher magnification in the Hfr. (**b**) Adherens junctions were visualized by N-Cadherin expression on PN2 and PN6 in both control and treated animals. On PN2 the slight changes in the apical localization of N-Cadherin are observed; misexpression of this marker is more pronounced on PN6. Confocal images were taken at the ventricle (Vn). (**c**) In control animals the stem/progenitor cell marker SOX2 is prominently expressed in the posterior ventricular epithelium on the day of birth (PN0). Visualization of blood vessel via PECAM-1 in this same section demonstrates the extensive vascular network that is normally present in the neonatal rat brain. High magnification images highlight the enrichment of the vasculature within the SOX2+ and Vimentin+ population. These cells are also in proximity to the Vn, which contains cerebrospinal fluid (CSF). Together, this progenitor niche resides at the intersection of TH transport (vasculature and CSF) in neonates. All scale bars represent 50 µm and N = 3–4 were analyzed.

Gene expression analyses on PN6 revealed alterations in *Pax6* (Fig. 5a’). Given the role of *Pax6* in the maintenance and regulation of radial glial progenitors^18^, we next examined radial glial cell morphology in a subset of littermates. Radial glial cells form a structural scaffolding that mediate neuroblast migration in the developing brain^23^, and alterations in these progenitors have been attributed to periventricular heterotopia formation in both rodents and humans^17,24–26^. Vimentin immunofluorescence on PN6 revealed significant changes in radial glial structure within the posterior forebrain of PTU treated animals. These differences were especially prominent in the heterotopia forming region, which is directly adjacent to the ventricular epithelium (Fig. 6a, white bounding box). In the control brain these progenitors possess their hallmark radial polarization; this is in direct contrast to the truncated, highly disorganized fibers observed in the brain of treated pups (Fig. 6a, high magnification).

As radial glial cells normally anchor to the ventricular epithelium, we next asked whether alterations in cell adhesion of this region coincided with the observed changes in these progenitor cells. On PN2, differences in the spatial expression of N-Cadherin are observed in the epithelium of treated animals (Fig. 6b). By PN6, the normal apical distribution of N-Cadherin is further disrupted in PTU-treated animals, suggesting that cell adhesion of the ventricular epithelium is altered under conditions of transient hypothyroidism. We propose that the failure of radial glial cells to anchor at the apical surface, due to disruption in cell adhesion, may underlie the observed changes in radial glial morphology (Fig. 6a).

### The heterotopia forms at the junction of multiple brain barriers

**Figure 7:**
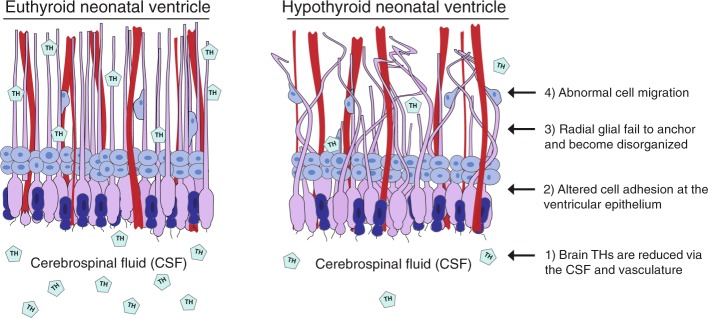
Hypothesized working model of heterotopia formation induced by developmental TH insufficiency. In the euthyroid neonate cells of the lateral ventricular neuroepithelium are a tightly organized progenitor population, which includes radial glial (lilac) and maturing neuroependymal cells (dark purple). Radial glial cells physically anchor to the apical surface of the ventricular zone via adherens junctions and possess cilia that extend into the cerebrospinal fluid (CSF). The CSF contains thyroid hormones (pictured here as TH). In addition to residing near the CSF, the neuroepithelium is also highly vascularized (red), which represents a second source of THs. In the hypothyroid pup decreased THs, via the CSF and the vasculature, lead to abnormal adherens junctions and loss of radial glia polarity. The marked changes in the radial glia scaffolding leads to abnormal neuronal migration and consequently, the periventricular heterotopia.

As the periventricular heterotopia is highly penetrant and in a reproducible anatomical location, characteristics of the ventricular epithelium may confer especial susceptibility to TH disruption. In the PN0 forebrain the ventricles are clearly open, including posteriorly where the heterotopia will later form (Fig. 6c, see “Vn”, and Supplementary Fig. 2). Visualization of neural progenitors in this region by SRY-Box 2 (SOX2) immunostaining, revealed an enriched population of SOX2+ cells within the ventricular epithelium of neonates (Fig. 6c, ventricular zone). The cerebrospinal fluid (CSF) is one source of brain T4/T3, and the CSF circulates throughout the ventricular system of the brain^27^. Thus, the apical surface of these predominantly SOX2+ cells is in direct contact with one source of THs. The vasculature represents the second source of brain THs; specifically T4/T3 are actively transported across the blood brain barrier (BBB) and into the tissue^27^. To determine if these SOX2+ cells were also in proximity to vasculature, endothelial cells of this region were detected by platelet-endothelial cell adhesion molecule 1 (PECAM-1) immunostaining. This experiment demonstrated that the posterior forebrain of neonates possesses an extensive vasculature network, with enriched PECAM-1 expression localizing to both the SOX2+ and Vimentin+ neuroepithelium (Fig. 6c, note co-staining). Thus, the ventricular neuroepithelium, marked by both SOX2+ and Vimentin+ progenitors, is directly abutting or near both the CSF and vasculature. Residence at this crucial intersection of hormone transport may render these cells especially sensitive to alterations in brain T4/T3 concentrations. This hypothesis is depicted in Fig. 7, our working model of heterotopia formation.

## Discussion

Here we show that pregnant rats exposed to a low dose of PTU for five days (GD19-PN2) induces a highly penetrant cortical heterotopia in their offspring. Given that rats give birth to relatively altricial young, this window of sensitivity correlates to the second trimester of human pregnancy. While analyses of THs in the serum and brain demonstrate that T3 is relatively resilient to perturbation, these data show a finite period of altered TH homeostasis is still sufficient for birth defect formation in pups. A second key finding of this study is that in contrast to its initial description as subcortical band, this defect is more accurately described as a periventricular heterotopia. This clarified definition, revealed by our temporal examination of heterotopia morphogenesis, has potentially important implications for the understanding of how different compartments of the brain may be especially sensitive to TH disruption.

Following the testing of several hypotheses to describe the developmental mechanism underlying heterotopia formation, alterations in radial glial morphology are likely a major contributing factor. Disruption of these scaffolding-like cells, and consequently normal cell migration, is attributed to various classes of cortical dysplasia in both animal models^17,24,28–31^ and humans^26,32^. While many of these works describe effects of germline mutations or transient genetic knockdowns, the work here demonstrates that a thyroid mediated mechanism is upstream of the observed morphological changes in these progenitors. Previous reports in rats and mice have established that TH disruption can alter radial glial structure in the cortex at various times across development^33–37^. While there is a strong weight of evidence to suggest that radial glia may be a target of THs, it has remained unclear why.

During normal neurodevelopment radial glia progenitors attach to the apical neuroepithelium via specialized endfeet; these endfeet anchor these cells, physically mediating their normal apico-basal polarity (reviewed in Chou *et al*.^23^. Thus, the structural integrity of the neuroepithelium directly contributes to the normal functioning of these cells. Concurrent with our identified alterations in radial glial morphology, we also detected changes in the spatial distribution of the adherens junction component N-cadherin within hypothyroid pups. Specifically, abnormalities were observed along the apical side of the epithelium, suggesting that cell-cell contact may be compromised in this region. While it is unclear from the data here if this is causative to the observed radial glia phenotype, others have shown that loss of neuroepithelial adherens junctions induce changes in radial glia structure and function, which can lead to cortical heterotopia^17,31,38,39^. With regards to thyroid signaling, differential expression of adherens junctions components like actin filaments and integrins have been reported in the brains of developing hypothyroid rodents^33,40–43^, and several other studies have shown that both T4 and reverse T3 (rT3) can directly modify cytoskeletal components via extranuclear signaling pathways^40,44–46^. Therefore dysregulation of TH signaling, even for a finite developmental period, may convergently affect cell junctions within the brain. These changes could manifest as various abnormalities in cytoarchitecture, including but not limited to abnormal adherens junctions and radial glial cells as we report here.

While this work does not identify a specific gene regulatory network leading to heterotopia formation, it does implicate pathways that may be regulated by THs at the ventricular epithelium. In our transcriptional analyses two genes associated with periventricular heterotopia in humans and rodents, filamin A (*FlnA*) and reelin (*Reln*), were not differentially expressed between hypothyroid and control animals (for review of genetic etiologies of heterotopia development see Liu *et al*.^47^ and Guerrini *et al*.^48^). We did observe a significant reduction in *Spred1*, a negative regulator of Ras/MAPK signaling. In a study by Phoenix *et al*., authors report that transient reduction of *Spred1* in the mouse brain results in *Pax6* upregulation, alterations in cell adhesion of the neuroepithelium, and loss of radial glia polarity. Remarkably, *Spred1* knockdown animals often exhibited periventricular heterotopia of the posterior forebrain^17^. Taken together many aspects of their data, both molecularly and phenotypically, directly parallel the data we report here. As SPRED1 functions as a multidomain scaffolding protein it is not unlike other structural proteins commonly associated with cortical dysplasia; we have identified that this gene may be modulated by THs. In addition to decreased expression of *Spred1*, we also observed differential regulation of several direct T3 targets, including a significant reduction in *Shh*. This morphogen is an established mediator of TH action in the brain^19–22^, and we showed pronounced SHH expression within the ventricular epithelium of control neonates. This expression pattern is reminiscent of *Spred1*, which is also enriched in the ventricular epithelium of late gestation mouse embryos^17^. Importantly, *Shh* downregulation was detected in the brains of hypothyroid pups on PN2. This reduction was one of the few changes identified at this early stage, preceding differential expression of both *Spred1* and *Pax6* on PN6. While it is recognized that SHH has many cellular roles, it has been implicated in the maintenance of cell adhesion *in vivo*^49,50^, suggesting that a similar mechanism could be occurring in our rat model. Taken together, it is possible that *Spred1* is regulated by SHH, or both SPRED1 and SHH are contributing to heterotopia formation by parallel mechanisms.

One of the other notable function of SHH is regulation of cell proliferation in the brain^51^; however, we did not find significant differences in the percent of Ki67+ cells in TH insufficient pups on either PN2 or on PN6. Interestingly, we did identify reduced *Casp3* expression on PN6, and confirmed this gene expression result by cleaved-CASP3 cell counts. Together these experiments show that transient developmental hypothyroidism *decreases* apoptosis at the ventricular epithelium relative to control animals. While the underlying mechanism of this observation is unclear, this likely contributes to the accumulation of heterotopic cells in PTU treated pups. As cell death is an essential mechanism to control morphogenesis, these results further demonstrate the breakdown of normal developmental processes localized to the ventricular epithelium. So while it is unclear precisely how THs are affecting developmental networks in this cell population, the interactions between THs, associated nuclear and extranuclear receptors, and morphogenetic pathways are areas of future investigation.

Given that transiently hypothyroid neonates exhibit (i) abnormalities in radial glia morphology, (ii) alterations in cell adhesion, and (iii) reduced apoptosis, we propose that the ventricular epithelium is acutely sensitive to changes in TH concentrations during the perinatal period. Specifically, we hypothesize that the acute sensitivity of this progenitor cell niche results from the spatial dynamics of hormone transport. As T4/T3 are not diffusible into brain tissue, they must be actively transported by membrane bound molecules like monocarboxylate transporter 8 (MCT8, gene *Slc16a2*) and/or solute carrier organic anion transporter family member 1C1 (OATP1C1, gene *Slco1c1*)^52^. These transporters reside on the interfaces of the “brain barriers” which include the BBB (endothelial cells), the blood-CSF barrier (choroid plexus), and the CSF-brain barrier (apical cells of the ventricular epithelium)^27,53^. The CSF-brain barrier is comprised of ciliated radial glia cells and/or ependymal cells, and these cells are in direct contact with the CSF containing T4/T3^53–55^. In the present study we confirmed that the ventricular epithelia in perinatal rat pups also possess an enriched vasculature network. As such, this crucial neural progenitor pool lies juxtaposed to the two sources of brain THs, the CSF and the vasculature. Although we confirmed that T4 and T3 were reduced in both the serum and the brain of treated pups, assessment of THs within the CSF would provide additional support for this hypothesis. However, others have documented that TH insufficiency reduces T4/T3 in the CSF in addition to the serum of adult rats^56^.

Our working model of heterotopia formation (Fig. 7) leads to a compelling question: if reduced serum T4 concentrations in developing rats does not induce a quantifiable heterotopia, does this mean the brain is unaffected? While we cannot currently answer this question, identifying how serum biochemical measures like TSH and T4 relate to neurodevelopmental consequences is critical for regulatory testing of chemicals *in vivo*. As changes in both ventricular structure and radial glial morphology appear causal to the heterotopia, it is likely that this anatomical phenotype is a symptom of greater cellular changes within the brain. Additionally, this study highlights several important considerations. First, timing is everything; our data show that while the heterotopia represents a tangible readout of progenitor cell dysfunction, it is solely dependent on TH insufficiency during the perinatal period. Serum TH concentrations must be assayed during this period to predict birth defect formation in rats. Undoubtedly TH insufficiency during other stages could result in other discrete neurodevelopmental effects, and absence of a heterotopia does not signify that the brain is unaffected by TH disruption. Furthermore, in both rodents^12–14^ and children^57,58^, heterotopias are associated with profound functional effects including epilepsy and learning disabilities. While these functional outcomes are also consequences of abnormal brain development, it is unclear how decreased THs induce these effects, and if these functional outcomes can be present independently of a heterotopia in rats. To delineate what range of TH insufficiency is adverse, further dose-response studies are necessary. Dose response information would contextualize (i) what magnitude of TH disruption leads to heterotopia formation, (ii) what quantifiable biomarkers are the best readouts of adverse neurodevelopment, and (iii) what functional assays best capture symptoms of TH insufficiency. This quantitative framework could also inform the clinical management of thyroid disease during pregnancy. Currently several epidemiological studies have identified TH requirements during early fetal development^4,59^, whereas the present study suggests that later developmental periods are also highly susceptible to TH perturbation. In the future it will be imperative to test associations between TH insufficiency during the last two trimesters of human pregnancy with specific neurodevelopmental outcomes in children. In total this proposed dose-response framework would aid in evaluating TH toxicants *in vivo* and could also serve as a valuable reference for the medical community.

## Conclusions

The presented results demonstrate that five days of goitrogen treatment during the perinatal period in pregnant rats is sufficient to induce a highly penetrant periventricular heterotopia in offspring. This period in rats coincides with the second trimester of human development. Our findings also show that the ventricular epithelium, a progenitor cell niche, may be acutely affected by TH insufficiency. This spatial susceptibility may be mediated by the juxtaposition of this population to the two sources of brain THs, the cerebrospinal fluid and vasculature. These results provide a cellular mechanism linking transient TH disruption to adverse brain development and suggest that certain developmental periods and sub-compartments of the brain may be especially sensitive to TH dysfunction.

## Materials and Methods

### Animal husbandry

All experiments were conducted with prior approval from the United States Environmental Protection Agency’s Institutional Animal Care and Usage Committee (IACUC) and were carried out in an Association for Assessment and Accreditation of Laboratory Animal Care (AAALAC) approved facility. All methods were carried out in accordance with the relevant guidelines and regulations. Timed pregnant Long Evans rats were purchased from Charles River (Morrisville, NC) and delivered on gestational day (GD) 6; sperm positive was considered GD0 and pup birth postnatal day (PN) 0. Dams were single housed in hanging cages, maintained on a 12:12 light cycle, and offered chow (Purina 5008) and deionized water *ad libitum*.

### Exposures to induce hypothyroidism

To determine the developmental period necessary for heterotopia formation in offspring, dams were treated with 10 ppm (0.001%) 6-propyl-2-thiouracil (PTU, purity ≥98%, Sigma) dissolved in the deionized drinking water during four different exposure windows. The first window represented the positive control period that ensured complete penetrance of a heterotopia as supported by previous work (PTU treatment from GD9-PN2)^12–14^. The remaining three windows were the gestational and perinatal period divided into thirds (treatment from GD9-GD14, GD14-GD19 and GD19-PN2). Control dams were administered vehicle only (deionized drinking water). On PN14, one male and one female pup were screened for heterotopia incidence (see below). Once the critical window for heterotopia formation was identified, a second cohort of pregnant animals was treated (10 ppm PTU or vehicle) during this specific developmental period (GD19-PN2) in order to determine the potential mechanism of heterotopia formation. This experimental design is depicted in Supplementary Fig. 3.

### Heterotopia detection and analysis

For quantifying heterotopia volume in PN14 animals, brains were immersion fixed in 4% paraformaldehyde pH 7.4 for 48 hours before vibratome sectioning coronally at 60 µm in Tris-buffered saline (TBS). Free floating immunohistochemistry was then performed to detect mature neurons by NeuN reactivity according to our previously published methods^14,15^. In short endogenous peroxidase activity was quenched in 0.9% hydrogen peroxide, and nonspecific binding inhibited with serum blocking solution (2% horse serum in 0.3% triton in TBS). Sections were then incubated overnight in anti-NeuN antibody (1:2500, Millipore MAB377), washed, and incubated in biotinylated secondary antibody (1:400, Vector). After subsequent washes, the tissue was exposed to avidin-biotin complex for signal amplification (Vector) and developed with diaminobenzidine (DAB, Sigma) according to the manufacturers protocol. Sections were then mounted serially on slides, dehydrated, and cover slipped. Imaging was performed on an Aperio slide scanner and heterotopia area quantified using Aperio’s Image Scope software (Leica Biosystems). If ectopic neurons were detected in the heterotopia forming region, the perimeter of the NeuN+ cluster was traced, and area calculated. From these area measures heterotopia volume was estimated by summing the total calculated heterotopia areas across sections, and then multiplying by both section thickness (60 µm) and interval according to described stereology procedures^60^. In the present study we defined a cortical heterotopia as a mass of neurons within the posterior forebrain that was ≥0.006 mm^3^.

### Serum thyroid hormone quantification

On PN2 dams were briefly restrained in a Tailveiner® (Braintree Scinetific), and blood collected from the tail vein for subsequent serum TH analysis. With regards to the developing animals, male and female pups from each litter were decapitated on PN0, PN2, and PN6, and whole trunk blood collected for serum TH analysis; if multiple littermates were sampled, then the blood was pooled as a single sample. All serum was isolated from whole blood via a serum separator tube with clot activator gel (Benton Dickinson) according to our previously published methods^14,61^. Isolated serum was aliquoted and stored at -80 °C until analysis. Total T4 and T3 were analyzed by mass spectrometry (LC/MS/MS) as previously described^14,61^. The lower limit of quantification (LLOQ) for both T4/T3 were 0.1 ng/ml. TSH was analyzed by radioimmunoassay according to previously published methods^62,63^.

### Brain thyroid hormone quantification

Pup brains on PN0, 2 and 6 were extracted from the skull, the cerebellum and olfactory bulbs removed, and the forebrain snap frozen in liquid nitrogen for analysis of tissue TH concentrations. All samples were stored at −80 °C until analysis. THs were isolated by solid phase extraction, and analyzed by LC/MS/MS as previously described^64^. The LLOQ for both total T4 and T3 in the brain tissue was 0.1 ng/g.

### Quantitative real-time PCR (qRT-PCR)

A section of forebrain including the heterotopia forming region was isolated from male pups (dams treated from GD19-PN2) to assay for gene expression changes on PN2 and PN6, and a schematic of this dissection is depicted in Supplemental Fig. 4. Following isolation, tissue was preserved in RNAlater™ (Invitrogen) according to the manufacturer’s recommendation. Total RNA was extracted via TRIzol® (Invitrogen) and samples were DNase treated (Promega) according to previously published methods. RNA quantity and purity were quantified via a Nanodrop™ (Thermofisher), and sample integrity measured using a Bioanalyzer 2100™ (Agilent). Only samples with a 260/280 ≥ 1.9 and a RNA-integrity number (RIN) ≥ 9 were used in downstream applications. cDNA was synthesized using random primers (Applied Biosystems), and subsequently diluted in nanopure water. Quantitative PCR (qPCR) was performed on a 7600HT Fast Real-Time PCR System (Applied Biosystems), using TaqMan Universal PCR Master Mix and commercially purchased TaqMan probes (Applied Biosystems). The thermocycler program was as follows: 95 °C for 5 minutes, followed by 40 cycles of 15 seconds at 95 °C, and 1 minute at 60 °C. Each sample was duplicated in duplicate, and the data analyzed using 2^−ΔΔct^ method with β2-microglobulin (B2M) as the reference gene. B2M was utilized as the reference based on its stability in our samples as assessed by NormFinder^65^. Information on all Taqman probes can be found in Supplementary Table 1.

### Immunohistochemistry

Pups were euthanized by an overdose of intraperitoneally administered Euthasol® on PN0, 2, 4, 6, and 14, and the brain preserved via cardiac perfusion. Briefly, the circulatory system was flushed with 0.1M PBS before fixation with 4% PFA pH 7.4. The brain was immediately extracted from the skull and postfixed in fresh 4% PFA overnight, and cryoprotected in 30% sucrose/PBS before embedding in Tissue Freezing Medium™ (Fisher Scientific). Brains were cryosectioned coronally at 35 µm and collected in PBS for free floating immunohistochemistry. For calorimetric detection, endogenous peroxidase was first inhibited by incubation in 0.3% hydrogen peroxide diluted in 100% methanol. Nonspecific binding was reduced by incubation in serum block (10% horse serum, 10% goat serum, 0.1% tween diluted in 0.1M PBS), and the signal amplified by use of avidin-biotin complex (ABC kit, Vector); the reaction was developed with DAB. Slides were then mounted, dehydrated, and cover slipped before imaging using an Aperio slide scanner (see above). For fluorescent detection, nonspecific binding was again reduced using the same serum block (10% horse serum, 10% goat serum, 0.1% tween diluted in 0.1M PBS) before overnight incubation with primary antibody. All primary antibodies were recognized with various Alexa Fluor secondary antibodies; all antibody vendors, concentrations, and combinations are referenced in Supplementary Table 2. Sections were then mounted, cover slipped using ProLong™ Diamond Antifade Mountant (Thermo Fisher) and imaged using a Nikon A1 laser scanning confocal microscope. Images were processed using NIS-Elements Advanced Research (Nikon).

### Cell birth dating and detection

A subset of control (N = 10) and treated dams administered PTU from GD19-PN2 (N = 10) were pulsed with 50 mg/kg of 5-ethynyl-2-deoxyuridine (EdU, Santa Cruz) on GD18 and GD19 to birthdate cells in the developing brain. EdU was dissolved in sterile 0.1M PBS and heated to 50 °C for approximately ten minutes until dissolved; animals were injected intraperitoneally with solution cooled to ≤35 °C. Labeled cells were detected using the Click-iT® EdU Alexa Fluor 568 Imaging kit (Thermo) according to the manufacturer’s protocol. Following the Click-iT reaction, tissue was then thoroughly washed in PBS before proceeding to immunohistochemistry as described above.

### Quantifying cell proliferation and death

Ki67+ and CASP3+ cells were analyzed in every 5^th^ section across a defined anterior-posterior interval which encompasses the heterotopia forming region. Using the Nuclear Cell Count Module in Aperio’s Image Scope, positively and negatively staining cells were quantified in a 20x bounding box encompassing the medial region of the epithelium of the lateral ventricles. All counts are expressed as a percentage of positively stained cells.

### Statistics

Litter was considered the biological replicate in all presented statistics. Body weights, serum THs, and brain THs were all analyzed using repeated measures ANOVA with an alpha level of 0.05. Gene expression was analyzed by ANOVA, and multiple testing corrected by reducing the alpha level to 0.01. Cell proliferation and death data were analyzed by two tailed independent t-tests with an alpha level of 0.05.

### Figure Preparation

All images were edited in Adobe Photoshop, and figures prepared in either Photoshop or Adobe Illustrator. Artwork was produced in Illustrator.

## Supplementary information

Supplementary Information

## Data Availability

The datasets generated during the current study are available via Science Hub. (https://sciencehub.epa.gov/sciencehub).
