# Supplementary material for: A transient window of hypothyroidism alters neural progenitor cells and results in abnormal brain development
Source: Sci Rep. 2019 Mar 15;9:4662. doi: 10.1038/s41598-019-40249-7 (PMC6420655; doi:10.1038/s41598-019-40249-7)
Supplement: Supplementary file 1 — Supplementary Information [file 41598_2019_40249_MOESM1_ESM.pdf]

A transient window of hypothyroidism alters neural progenitor cells and results in abnormal brain development

Katherine L. O'Shaughnessy, Susan E. Thomas, Stephanie R. Spring,
Jermaine L. Ford, Richard L. Ford, and Mary E. Gilbert

Supplementary Information

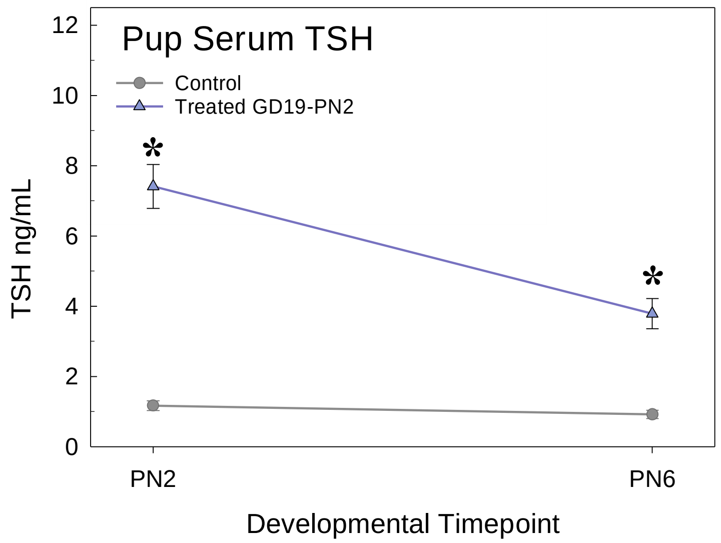

Supplementary Figure 1. Thyroid stimulating hormone (TSH) in early postnatal pups. PTU treatment significantly increased serum TSH concentrations in animals born to dams treated with PTU from GD19-PN2. TSH was not assayed on PN0 due to limitations in sample volume. Asterisks represent $p < 0.05$.

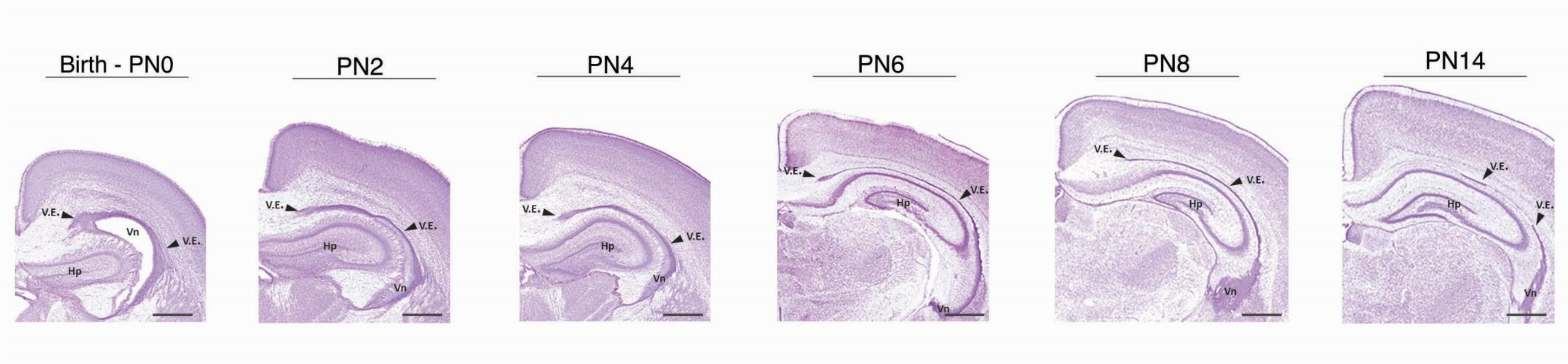

Supplementary Figure 2. Developmental ontogeny of the ventricular epithelium of the posterior forebrain. On the day of birth (PN0), the lateral ventricles (Vn) are clearly open, surrounded by a pronounced epithelium. While the ventricles remain slightly open from PN2-PN14, the ventricular epithelium (V.E.) appears to condense as the animal develops. Hp= hippocampus and scale bar = 500 μm.

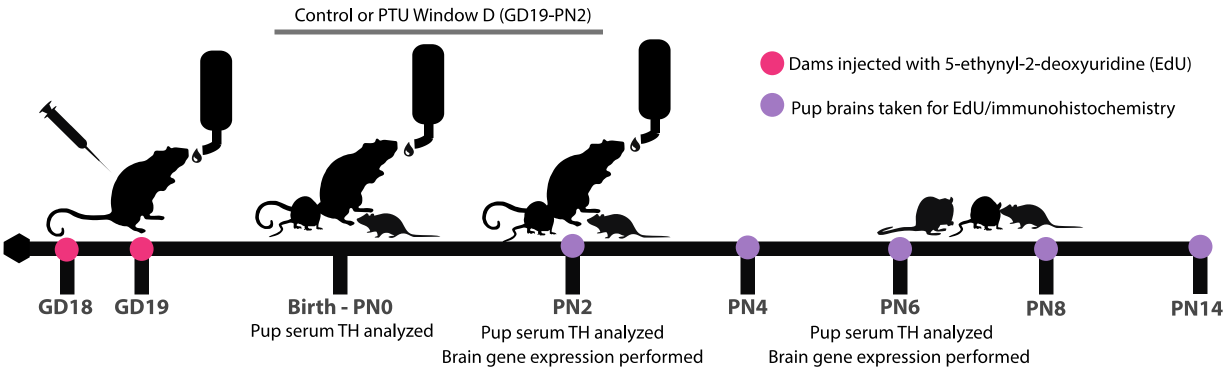

Supplementary Figure 3. Experimental design for determining the developmental basis of heterotopia formation. As maternal PTU exposure from GD19-PN2 induces complete penetrance of the heterotopia in offspring, this acute treatment for birth dating analysis.

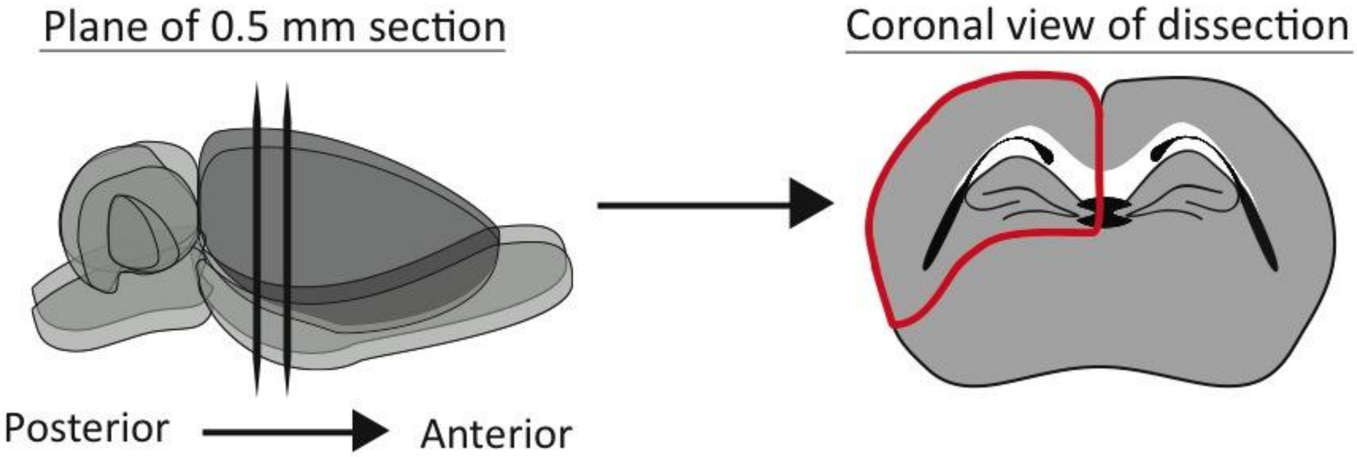

Supplementary Figure 4. Dissection method for gene expression analyses. On PN2 and PN6 brains from control and treated pups were immediately extracted from the skull. A 0.5 mm section was sliced from the posterior forebrain, and the heterotopia forming region was isolated as depicted (red area).

Gene name	Gene Symbol	NCBI Gene ID	TaqMan Primer
Thyroid receptor alpha	*Thra* (TRα)	81812	Rn01464140_m1
Thyroid receptor beta	*Thrb* (TRβ)	24831	Rn00562044_m1
Deiodinase II	*Dio2*	65162	Rn00581867_m1
Monocarboxylate transporter 8	*Slc16a2* (MCT8)	259248	Rn00596041_m1
Organic anion transporter polypeptide 1C	*Slco1c1* (OATP1C1)	84511	Rn00584891_m1
Hairless	*Hr*	60563	Rn00577605_m1
Kruppel like factor 9	*Klf9*	117560	Rn00589498_m1
Calcium/calmodulin-dependent protein kinase type IV	*Camk4*	25050	Rn00664802_m1
Paired box 8	*Pax8*	81819	Rn00579743_m1
Bone morphogenetic protein 7	*Bmp7*	85272	Rn01528889_m1
SRY-box 2	*Sox2*	499593	Rn01286286_g1
Doublecortin	*Dcx*	84394	Rn00670390_m1
Satb2	*Satb2*	501145	Rn01438160_m1
Sonic hedgehog	*Shh*	29499	Rn00568129_m1
Sprouty-related EVH1 domain containing 1	*Spred1*	296072	Rn01486390_m1
Prepronociceptin	*Pnoc*	25516	Rn01637101_m1
Paired box 6	*Pax6*	25509	Rn00689608_m1
Filamin A	*Flna*	293860	Rn01187530_m1
Reelin	*Reln*	24718	Rn00589609_m1
Roundabout guidance receptor 1	*Robo1*	58946	Rn00573395_m1
Slit guidance ligand 2	*Slit2*	360272	Rn00575268_m1
Brain derived neurotophic factor total	*BDNF-t*	24225	Rn02531967_s1
Nerve growth factor	*Ngf*	310738	Rn01533872_m1
Caspase 3	*Casp3*	25402	Rn00563902_m1
Beta-2-microglobulin	*B2M*	24223	Rn00560865_m1

Supplementary Table 1. Probes utilized in gene expression analyses.

Primary Antibody	Vendor	Catalog Number	Dilution	Secondary Antibody	Vendor	Catalog Number	Dilution
Anti-NeuN	Millipore	MAB377	1:2500, calorimetric	Biotinylated anti-mouse and ABC kit	Vector	PK-6102	1:400
Anti-NeuN	Millipore	MAB377	1:300, fluorescence	Goat Anti-Mouse Alexa 488	Abcam	ab150117	1:400
Anti-SHH	Santa Cruz	sc-365112	1:500, calorimetric	Biotinylated anti-mouse and ABC kit	Vector	PK-6102	1:400
Anti-Vimentin	Abcam	ab24525	1:250, fluorescence	Goat Anti-Chicken Alexa 647	Abcam	ab150175	1:400
Anti N-Cadherin	Santa Cruz	sc-271386	1:400, fluorescence	Goat Anti-Mouse Alexa 488	Abcam	ab150117	1:400
Anti-Sox2	Abcam	ab97959	1:300, fluorescence	Donkey Anti-Rabbit Alexa 555	Abcam	ab150066	1:400
Anti-PECAM-1	Santa Cruz	sc-376764	1:50, fluorescence	Goat Anti-Mouse Alexa 488	Abcam	ab150117	1:400
Anti-Cleaved Casp3	Cell Signaling	9661S	1:1000, calorimetric	Biotinylated anti-rabbit and ABC kit	Vector	PK-6101	1:400
Anti-Ki67	Abcam	ab16667	1,1000 calorimetric	Biotinylated anti-rabbit and ABC kit	Vector	PK-6101	1:400

Supplementary Table 2. Antibody combinations used in this study. Each row represents an immunohistochemistry protocol employed in this study; all antibody vendors, catalog numbers, and concentrations are listed.
